# Single-Cell Profiling Identifies a CCR2^+^ Neutrophil-like Population Associated with Colorectal Cancer Liver Metastasis in a Murine Model

**DOI:** 10.3390/genes17070831

**Published:** 2026-07-21

**Authors:** Zi-Jun Yan, Yuan-Jie Yin, Yu-Ting Wang, Xian-Qi Zhang, Xiong-Hui Wang, Xi Chen, Cai-Ning Zhao, Rong Liu

**Affiliations:** 1 Faculty of Hepato-Biliary-Pancreatic Surgery, The First Medical Center of Chinese People’s Liberation Army (PLA) General Hospital, Beijing 100036, China; 2 Department of Clinical Oncology, School of Clinical Medicine, Li Ka Shing Faculty of Medicine, The University of Hong Kong, Hong Kong SAR 999077, China; 3 Xiangya School of Basic Medical Sciences, Central South University, Changsha 410075, China

**Keywords:** colorectal liver metastases, CCR2, neutrophil, myeloid cell, single-cell RNA sequencing

## Abstract

Background/Objectives: Colorectal liver metastases (CRLMs) are a major contributor to recurrence and mortality in colorectal cancer (CRC), with approximately a quarter of patients developing liver metastases over the course of the disease. Bone-marrow-derived myeloid lineages are sent into the circulatory system and colonize pre-metastatic niches, yet the transcriptional programs by which they establish a pro-metastatic microenvironment remain incompletely defined. Methods: Using an MC38 splenic-injection CRLM mouse model, we generated single-cell RNA sequencing (scRNA-seq) profiles of FACS-sorted CD11b^+^Gr1^+^ bone marrow myeloid cells, together with bulk RNA sequencing profiles of bone marrow and peripheral blood. Downstream analyses were performed in silico, including clustering and annotation, trajectory inference, cell–cell communication analysis, weighted gene co-expression network analysis (WGCNA), and pathway enrichment, with subset specificity examined against a public dataset of *E. coli* (*Escherichia coli*)-infected mice. Results: Within the CD11b^+^Gr1^+^ compartment, a CCR2^+^ neutrophil-like population (*Ly6g*^+^*S100a8/9*^+^) emerging during terminal differentiation was identified, which was enriched in CRLM mice but nearly absent in controls. Communication inference revealed an FN1-CD44 interaction involving mature neutrophils, which was associated with an epithelial–mesenchymal transition signature and upregulation of *Tgfb1* and *Il1b*. This subpopulation was not recovered in the infection dataset, suggesting relative specificity to CRLMs. Conclusions: Within the constraints of a splenectomized hepatic colonization model, integrated transcriptomic analysis highlighted a CCR2^+^ bone marrow neutrophil-like population as a candidate contributor to CRLM, challenging the view that CCR2^+^ pro-metastatic myeloid cells are exclusively monocytic and suggesting candidate biomarkers and therapeutic targets for further study.

## 1. Introduction

Colorectal liver metastases (CRLMs) are a major determinant of prognosis in colorectal cancer (CRC) patients [1]. The liver is the most common distant metastatic site in CRC, and approximately 25–30% of CRC patients develop liver metastases over the course of the disease [2]. Current treatment strategies include surgical resection, radiofrequency ablation, chemotherapy, and biologic or immunotherapeutic approaches [3]. Despite these advances, the majority of patients with liver metastases eventually experience recurrence and disease progression, underscoring the urgent need for novel therapeutic strategies.

The liver is an immunologically tolerogenic organ with complex immune activity, which may contribute to its high susceptibility to metastatic colonization [4], particularly in gastrointestinal cancers such as CRC [5,6] and pancreatic cancer [7], as well as melanoma [8] and lung cancer [9,10]. Within this unique immune microenvironment, myeloid populations play critical roles in shaping pre-metastatic and metastatic niches [11]. CD11b^+^Gr1^+^ cells represent a heterogeneous myeloid population comprising both granulocytic and monocytic lineages [12,13]. Derived from granulocyte–monocyte progenitors (GMPs), these cells are critical components of the tumor immune microenvironment (TIME) and have been implicated in tumor metastasis and recurrence [14,15].

CRLM develops through a continuous, multistage process, progressing from latent micro-metastasis to overt macro-metastasis. During this process, myeloid lineages are moved from the bone marrow into the circulatory system and recruited to pre-metastatic niches [16] in response to chemokine signaling [17,18], thereby establishing an environment conducive to tumor progression through suppression of anti-tumor immunity [19] and promotion of tumor metastasis [20]. Importantly, myeloid recruitment is not uniform across metastatic contexts. For instance, CCL2/CCR2-dependent recruitment of CD11b^+^Gr1^mid^ myeloid cells has been reported to promote CRLM but not melanoma liver metastasis, suggesting tumor-context-specific regulation of myeloid trafficking [14]. However, CD11b^+^Gr1^+^ cells comprise heterogeneous granulocytic and monocytic populations, and the precise lineage identity, ontogeny, and functional specialization of CCR2-responsive myeloid cells in CRLM remain incompletely understood.

Chemokine-guided myeloid trafficking is a key determinant of metastatic niche formation. Among these pathways, the CCL2/CCR2 axis has been repeatedly implicated in CRC dissemination and liver metastasis. CCR2 (C-C motif chemokine receptor 2) is canonically associated with the recruitment of monocytes, macrophages, and monocytic myeloid-derived suppressor cells (Mo-MDSCs) [4]. In CRC, dormant CCR2^+^ metastatic CRC cells can respond to CCL7 secreted by Mo-MDSCs to drive metastatic outgrowth via the JAK/STAT3 pathway [21], whereas CCR2^+^ tumor-associated macrophages (TAMs) are recruited from the bone marrow in response to tumor-derived CCL2 [22]. TCF4-driven activation of the CCL2/CCR2 pathway further promotes macrophage recruitment and M2-like polarization at metastatic sites [23]. Together, these findings support the notion that there is an important role for CCR2-dependent interactions between metastatic CRC cells and immunosuppressive myeloid cells [24].

However, most studies of CCR2 in CRC metastasis have focused on monocytic cells, macrophages, or tumor cells themselves. This monocyte-centered view may overlook the heterogeneity of CD11b^+^Gr1^+^ myeloid populations, particularly the neutrophil lineage. Although CCR2 is not conventionally regarded as a neutrophil-associated chemokine receptor, emerging evidence indicates that neutrophils can acquire functional CCR2 under inflammatory and pathological conditions, enabling their mobilization from the bone marrow and recruitment to peripheral tissues [25,26]. Given the growing recognition of neutrophil heterogeneity in cancer [27], whether CCR2 marks a distinct pro-metastatic neutrophil subset in CRLM and how such cells contribute to liver metastatic niche formation remain largely unexplored and warrant further investigation.

We hypothesized that the CCR2^+^ myeloid compartment in CRLM may not be exclusively monocytic and could include a distinct neutrophil-like population with transcriptional features potentially associated with metastatic niche formation. The primary outcomes of this study were to identify and characterize this population by scRNA-seq, define its inferred differentiation trajectory, examine predicted intercellular communication with mature neutrophils, and identify circulating transcriptomic programs associated with CRLM progression. To address this question, we leveraged single-cell RNA sequencing (scRNA-seq) to resolve the heterogeneity of bone marrow CD11b^+^Gr1^+^ cells in an MC38 intrasplenic injection model of CRLM. We identified a CCR2^+^ neutrophil-like population characterized by a CD11b^+^Gr1^+^Ly6G^hi^ phenotype that was enriched in CRLM-bearing mice. Through integrated single-cell and bulk transcriptomic analyses, cell–cell communication inference identified a predicted FN1–CD44 interaction between CCR2^+^ neutrophil-like cells and mature neutrophils, which was associat-ed with transcriptional activation of the epithelial–mesenchymal transition (EMT) program [28,29]. Moreover, peripheral blood transcriptomes further implied that JAK-STAT and TGF-β signaling were associated with enrichment of the CCR2^+^ neutrophil-like population [30]. Together, these transcriptome-derived findings highlight this population as a candidate contributor to CRLM and provide a basis for future functional investigation. Defining this population may revise the current monocyte-centric understanding of CCR2 signaling in CRLM and reveal an unrecognized neutrophil-mediated mechanism of metastatic progression.

## 2. Materials and Methods

### 2.1. Mouse Model, Sample Collection, and FACS Sorting

Six-week-old male C57BL/6J mice were obtained from Shanghai Model Organisms Center (Shanghai, China). All animal experiments and procedures were conducted in accordance with the Guide for the Care and Use of Laboratory Animals of the National Institutes of Health. The study protocol was reviewed and approved by the Ethics Committee of the Scientific Investigation Board of the Second Military Medical University (Approval No. 2022-011). CRLM was established in 8 mice per group by intrasplenic injection of 2 × 10^5^ MC38 cells followed by splenectomy. Briefly, mice were anesthetized with ketamine (100 mg/kg, i.p.) and xylazine (10 mg/kg, i.p.), the spleen was exposed, and tumor cells suspended in 100 µL PBS were injected; after 2 min, the spleen was removed to prevent residual cells from proliferating at the injection site, and the abdominal wall was sutured in layers. Control mice underwent splenectomy at the same time and received an equal volume of PBS. During necropsy, livers were collected and photographed to document gross tumor formation. Liver weight and body weight were recorded, and the liver/body weight ratio was calculated as a gross surrogate measure of hepatic tumor burden. In total, for scRNA-seq, bone marrow samples of one CRLM mouse and one control mouse were used. For bulk RNA-seq, 15 samples were analyzed, comprising 4 bone marrow and 4 blood samples from CRLM mice and 3 bone marrow and 4 blood samples from sham mice. Animals were euthanized 14 days post-surgery, and bone marrow and peripheral blood were collected for single-cell and bulk RNA sequencing. Bone marrow cells were harvested by flushing femurs and tibias with RPMI-1640 supplemented with 2% fetal bovine serum, and erythrocytes were removed with ACK lysis buffer (Gibco, Thermo Fisher Scientific, catalog no. A1049201). To isolate the CD11b^+^Gr1^+^ compartment, cells were stained with fluorophore-conjugated antibodies against CD45 (APC-Cy7, catalog no. 557659, BD), CD11b (FITC, catalog no. 557396, BD), and Gr1 (APC, catalog no. 553129, BD); centrifuged at 400× *g*; and resuspended in 300 µL of PBS. Bone marrow cells were first gated based on FSC-A and SSC-A to exclude debris, followed by singlet discrimination using FSC-A and FSC-H and selection of live cells. CD45^+^ leukocytes were then selected, and CD11b^+^Gr1^+^ myeloid cells were gated within the CD45^+^ population as the parent population. The CD45^+^CD11b^+^Gr1^+^ cells were isolated by fluorescence-activated cell sorting (BD FACSCelesta flow cytometer, BD Biosciences, San Jose, CA, USA) and processed for scRNA-seq library preparation.

### 2.2. ScRNA-Seq Data Processing

Raw sequencing data generated on a NovaSeq 6000 (Illumina) were processed with CellRanger (v3.1.0). BCL files were converted to FASTQ and aligned to the mouse genome (mm10). Downstream analyses were performed using Seurat (v4.0.2) [31]. Cells with 100–5500 genes and mitochondrial fraction below 15% were retained. Doublet detection was performed on each sample prior to integration using DoubletFinder (v2.0), identifying 1029 doublets in the control sample and 1115 in the CRLM sample. All doublets were removed prior to integration, with 12,688 and 15,288 singlets retained, respectively. The 15,288 model-group and 12,688 control-group cells were integrated into a single object using FindIntegrationAnchors and IntegrateData and then normalized and scaled with NormalizeData and ScaleData, using the Wilcoxon rank-sum test with Bonferroni correction for multiple comparisons. Marker genes were defined by a minimum detection fraction of 25% (min.pct = 0.25) and a log2FC threshold of 0.25; DEGs were further filtered at an adjusted *p*-value < 0.05 and |avg_log2FC| > 1. The top 2000 variable features were used for RunPCA, and the first 20 principal components (PCs), selected by JackStrawPlot and ElbowPlot, were used for clustering and UMAP at a resolution of 0.8. Cell types were annotated using the CellMarker database, SingleR (v1.4.1), and published markers. For neutrophil sub-clustering, the first 5 PCs were used at a resolution of 0.5.

### 2.3. Pseudotime Trajectory and RNA Velocity Analysis

Differentiation trajectories were constructed with Monocle (v2.18.0) [32] by ordering cells over pseudotime using DEGs from the Seurat FindAllMarkers function. Pseudotime-dependent gene kinetics were visualized as BEAM heatmaps, and marker-gene trends were plotted per cluster. RNA velocity was estimated with scVelo (v0.2.4) [33], which uses spliced and unspliced mRNA counts to infer the prospective state of individual cells [34]; gene-specific transcription, splicing, and degradation rates were inferred and projected as velocity vectors on the UMAP.

### 2.4. Differential Expression and Functional Enrichment

ScRNA-seq DEGs within clusters were identified with the Seurat FindMarkers and FindAllMarkers functions, and bulk RNA-seq hub genes were derived from significant WGCNA modules. GO enrichment of CCR2^+^ neutrophil DEGs was performed and visualized with GOplot (v1.0.2), assessing biological processes, molecular functions, and cellular components at *p* < 0.05. Hub genes and DEGs from key modules were analyzed with ClueGO (v2.5.8) and CluePedia (v1.5.8) in Cytoscape (v3.8.0) against KEGG and the GO Biological Process, Cellular Component, Molecular Function, and Immune System Process categories, with enrichment evaluated by the Bonferroni step-down method (*p* < 0.05). For pathway-level comparisons, HALLMARK gene sets were assessed by GSEA and visualized with pheatmap (v1.0.12), and GSVA (v1.38.2) [35] with msigdbr (v7.4.1) was used to compare KEGG pathways (C2 curated gene sets) between CRLM and sham blood samples.

### 2.5. Cell–Cell Communication Analysis

Intercellular ligand–receptor communication among CD11b^+^Gr1^+^ myeloid states was inferred with CellChat (v1.1.3) [36] for the model and control groups. Incoming and outgoing signaling patterns and relative pathway intensities were quantified from ligand–receptor expression, and overall communication was compared between groups. The CCL and FN1 signaling pathways, which showed significant activity, were selected for detailed analysis of the interaction between the CCR2^+^ neutrophil-like population and MatureNeu.

### 2.6. Comparison with an External Infection Dataset

To assess whether the CCR2^+^ neutrophil state arises under inflammatory conditions, we retrieved scRNA-seq data on bone marrow CD11b^+^Gr1^+^ cells from *E. coli*-infected mice (GSE137539) [13] using GEOquery (v2.58.0), including samples GSM4081545, GSM4081546, GSM4081549, and GSM4081550. These data were processed with the same parameters as were used for the in-house dataset.

### 2.7. Bulk RNA-Seq and WGCNA

Co-expression networks were constructed from the bulk RNA-seq expression matrix using WGCNA (v1.70-3) [37]. Pairwise Pearson correlations were computed and transformed into a weighted adjacency matrix using a soft-threshold power of 8, selected to approximate scale-free topology. TOM and its dissimilarity (1-TOM) were used to cluster co-expressed genes into modules, visualized as a hierarchical dendrogram, with minimum module size of 50, maximum module size of 6000, and mergeCutHeight of 0.4. Module membership (MM) and gene significance (GS) were calculated to relate modules to sample groups, and hub genes (|MM| > 0.75 and |GS| > 0.2) were retained for downstream analysis and visualization. Module–trait correlations and hub gene significance were evaluated with the Benjamini–Hochberg false-discovery rate (FDR) correction.

### 2.8. Flow-Cytometric Analysis of Bone Marrow Cells

For flow cytometry validation, 6 mice per group were used. Bone marrow cells were harvested from CRLM and control mice by flushing femurs and tibias with RPMI-1640 medium supplemented with 2% fetal bovine serum. Erythrocytes were removed using ACK lysis buffer. Single-cell suspensions were stained with viability dye and fluorophore-conjugated antibodies against CD45, CD11b, Gr1, Ly6G, and CCR2. The following viability dye and antibodies were used: Fixable Viability Stain 510 (BD Biosciences, cat. no. 564406), anti-CD45-APC-Cy7 (BD Biosciences, cat. no. 557659), anti-CD11b-FITC (BD Biosciences, cat. no. 557396), anti-Gr1-APC (BD Biosciences, cat. no. 553129), anti-Ly6G-PE (BD Biosciences, cat. no. 551461), and anti-CCR2-PE-Cy7 (Biolegend, cat. no. 150612). After staining, cells were washed and resuspended in 300 µL PBS for flow-cytometric acquisition on a BD FACSCelesta flow cytometer. Bone marrow cells were first gated based on FSC-A and SSC-A to exclude debris, and then singlet discrimination was performed. FVS510-positive dead cells were excluded, and viable cells were used for subsequent analysis. CD45^+^ leukocytes were then selected, and this was followed by gating of CD11b^+^Gr1^+^ myeloid cells as the parent population, consistent with the FACS sorting strategy used for scRNA-seq. Within the CD45^+^CD11b^+^Gr1^+^ compartment, Ly6G and CCR2 expression was assessed, and Ly6G^+^CCR2^+^ double-positive cells were identified and quantified. The same gating strategy was applied to all samples. Flow cytometry data were analyzed using FlowJo software (v 10.8.1).

### 2.9. Statistical Analysis

Statistical analyses were performed in GraphPad Prism (v8.0.1), R (v4.0.5), and Python (v3.8.10), with visualization using ggplot2 (v3.3.5) and seaborn (v0.11.2). Two-group comparisons used a two-tailed Student’s *t*-test, and three-group comparisons used one-way ANOVA, with *p* < 0.05 considered statistically significant. Specific statistical tests, correction methods, and thresholds for scRNA-seq and bulk RNA-seq analyses are described in the corresponding subsections above.

## 3. Results

### 3.1. Single-Cell Atlas of Bone Marrow CD11b^+^Gr1^+^ Lineages in CRLM

**Figure 1:**
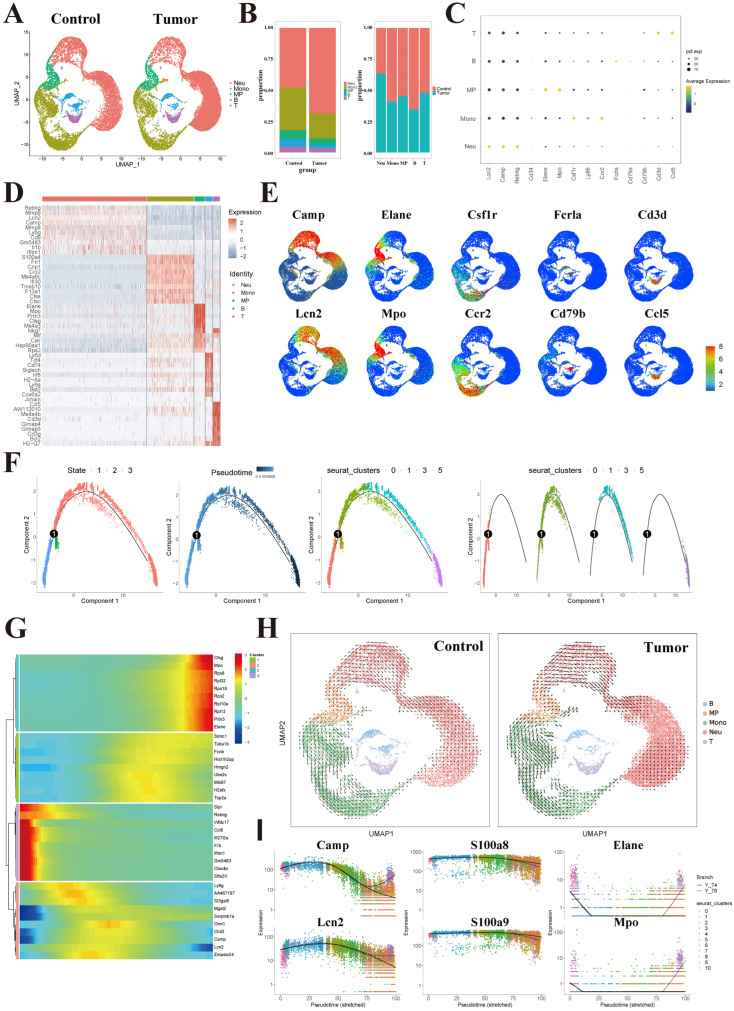
**Single-cell RNA-seq atlas of bone marrow Gr1^+^ myeloid lineages from MC38 intrasplenic-injection CRLM mice.** (**A**) UMAP of 30,196 high-quality cells colored by cell type; (**B**) proportion of cell types per sample (left) and samples per cell type (right); (**C**) dot plot of canonical markers for cell-type annotation, with dot size indicating percentage of expressing cells and color reflecting mean expression; (**D**) heatmap of the top 10 DEGs per cell type (Wilcoxon rank-sum test, Bonferroni-adjusted *p* < 0.05, |avg_log_2_FC| > 0.25); (**E**) UMAP feature plots of curated feature-gene expression; (**F**) Monocle pseudotime trajectory from myeloid progenitors to neutrophils; (**G**) BEAM heatmap of gene-expression dynamics over pseudotime (likelihood ratio test, q < 0.05); (**H**) RNA velocity projected onto the UMAP; and (**I**) expression dynamics of representative genes over pseudotime. CRLM, colorectal liver metastasis; DEGs, differentially expressed genes; UMAP, Uniform Manifold Approximation and Projection; BEAM, branched expression analysis modeling.

Bone marrow CD11b^+^Gr1^+^ cells from mice subjected to intrasplenic injection of MC38 cells were sorted by FACS and profiled by scRNA-seq. After quality control, 15,288 and 12,688 high-quality cells were retained from the model and control groups, respectively, with 17,330 genes detected in total. Graph-based UMAP clustering resolved eight clusters, which were annotated using SingleR and canonical markers and assigned to five populations, i.e., neutrophils (Neu—*Camp* and *Lcn2*), myeloid progenitors (MPs—*Elane* and *Mpo*), monocytes (Mono—*Csf1r* and *Ccr2*), B cells (*Fcrla* and *Cd79b*), and T cells (*Cd3d* and *Ccl5*) (Figure 1A,C,E), with cluster proportions shown in Figure 1B and integrated clustering on the top 10 variable genes shown in Figure 1D. Mobilization of CD11b^+^Gr1^+^ cells in CRLM was reflected by a greater abundance of neutrophils than monocytes. A distinct neutrophil group emerged in the model group but was nearly absent in the control. It showed elevated *Ccr2* expression while retaining myeloid-progenitor markers and was designated a CCR2^+^ neutrophil-like population.

### 3.2. A CCR2^+^ Neutrophil-like Population Emerges Along the Neutrophil Differentiation Trajectory

Monocle pseudotime analysis of MPs and neutrophils placed the CCR2^+^ neutrophil-like population at an advanced differentiation stage, closely aligned with mature neutrophils and arising at a distinct branch point as MPs progressed through intermediate states (Figure 1F). Pseudotime-ordered genes were resolved into four categories (Figure 1G): MP markers (*Mpo*, *Ctsg*, and *Elane*) peaked earliest, followed by proliferation genes (*Stmn1* and *Mki67*); neutrophils then transitioned from Ly6G^−^ to Ly6G^+^ with upregulation of *Lcn2* and *Camp*, and terminal mature neutrophils expressed *Ccl6*, *Il1b*, and *Retnlg* (Figure 1I). Mature-neutrophil markers (*Lcn2*, *Camp*, and *S100a8/9*) rose and stabilized, whereas *Elane* and *Mpo* declined to near-undetectable levels but rose sharply at the branch point, indicating this cluster was the CCR2^+^ neutrophil-like population. RNA velocity (Figure 1H) was consistent with bidirectional MP differentiation into neutrophils and monocytes; velocity vectors near the CCR2^+^ neutrophil-like population were shorter in the CRLM group than in the controls, suggesting altered cell-state dynamics in the tumor context.

### 3.3. CCR2^+^ Neutrophil-like Population Forms a Distinct Terminal State with Proliferative and Biosynthetic Features

**Figure 2:**
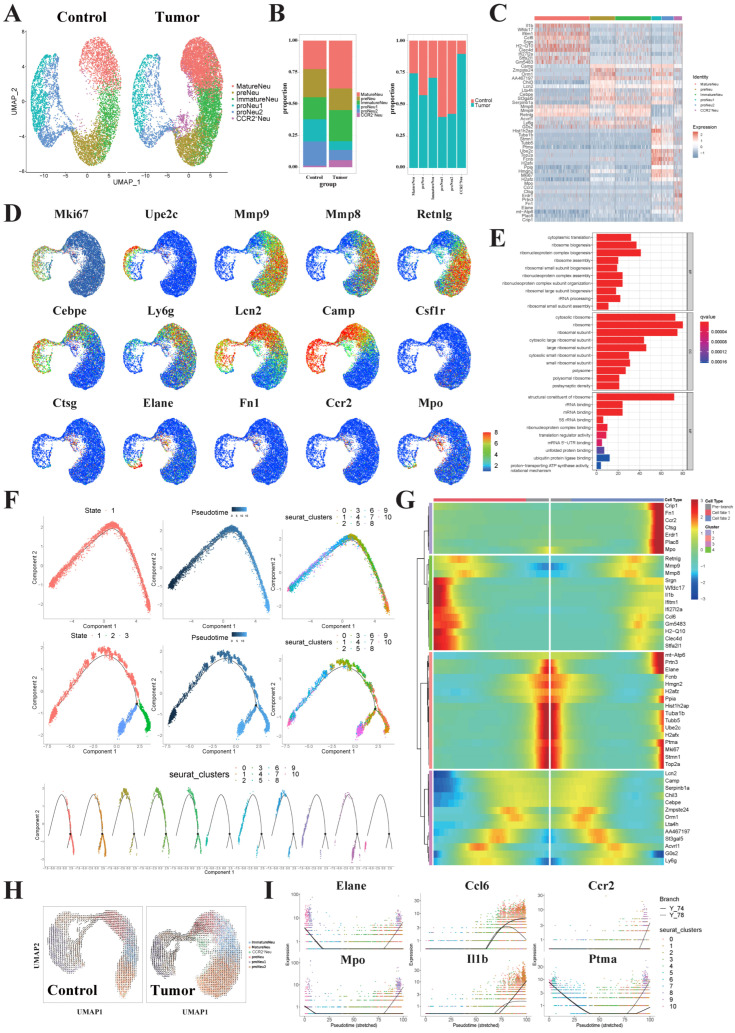
**Single-cell profiling of neutrophil subclusters within bone marrow Gr1^+^ myeloid lineages.** (**A**) UMAP of neutrophil subclusters by state; (**B**) proportion of neutrophil states per sample (left) and samples per state (right); (**C**) heatmap of the top 10 DEGs per state in the model group (Wilcoxon rank-sum test, Bonferroni-adjusted *p* < 0.05, |avg_log_2_FC| > 0.25); (**D**) UMAP feature plots of curated feature-gene expression in the model group; (**E**) GO enrichment of CCR2^+^ neutrophil DEGs showing the top 10 terms for molecular functions, biological processes, and cellular components (Fisher’s exact test, *p* < 0.05); (**F**) Monocle pseudotime trajectories for model and control groups from proNeu1/proNeu2 to MatureNeu, with the following state annotations: proNeu1 (subclusters 5 and 10), proNeu2 (subclusters 6 and 8), preNeu (subcluster 7), ImmatureNeu (subclusters 2–4), MatureNeu (subclusters 0 and 1), and CCR2^+^ neutrophil-like population (subcluster 9); (**G**) BEAM heatmap of differentiation dynamics in the model group (likelihood ratio test, q < 0.05); (**H**) RNA velocity projected onto the UMAP, with arrows indicating the predicted transcriptional state and confirming the differentiation trajectory; and (**I**) expression dynamics of representative genes over pseudotime.

Sub-clustering of the neutrophil compartment, following the established precursor–immature–mature continuum [12,13,27], grouped the 11 clusters into six states: proNeu1, proNeu2, preNeu, ImmatureNeu, MatureNeu, and CCR2^+^ neutrophil-like populations (Figure 2A). The CRLM group showed a higher proportion of MatureNeu and lower proportions of proNeu1/proNeu2, indicating a shift toward advanced differentiation (Figure 2B). Among the top 10 highly variable genes (Figure 2C,D), proNeu1/proNeu2 expressed *Cebpe* and *Stmn1*; ImmatureNeu/MatureNeu expressed *Mmp8*, *Mmp9*, and *Retnlg*; MatureNeu preferentially expressed *Ccl6* and *Il1b*; and the CCR2^+^ neutrophil-like population uniquely expressed *Mpo*, *Ccr2*, *Ctsg*, and *Fn1*. GO enrichment of CCR2^+^ neutrophil genes highlighted ribosome assembly (GO 0005840), cytoplasmic translation (GO 0002181), rRNA binding (GO 0019843), mRNA binding (GO 0003729), and positive regulation of the cell cycle (GO 1901989) (Figure 2E), indicating a transcriptionally and biosynthetically active state. Pseudotime ordering placed the CCR2^+^ neutrophil branch predominantly in the CRLM group (Figure 2F): neutrophils progressed from proNeu1/proNeu2 through preNeu, ImmatureNeu, and CCR2^+^ neutrophil-like populations to MatureNeu. The BEAM heatmap showed shared pre-branch genes for this population and MatureNeu that diverged thereafter (Figure 2G), with the CCR2^+^ neutrophil-like population specifically upregulating *Fn1*, *Ccr2*, *Ctsg*, and *Mpo* alongside the proliferation markers *Mki67* and *Stmn1*. RNA velocity (Figure 2H) indicated that, unlike in the controls, this population did not align with a single subgroup but represented an alternative terminal state predicted to interact with MatureNeu (Figure 2I).

### 3.4. Inferred FN1-CD44 and CCL6 Communication Between CCR2^+^ Neutrophil-like Population and Mature Neutrophils

**Figure 3:**
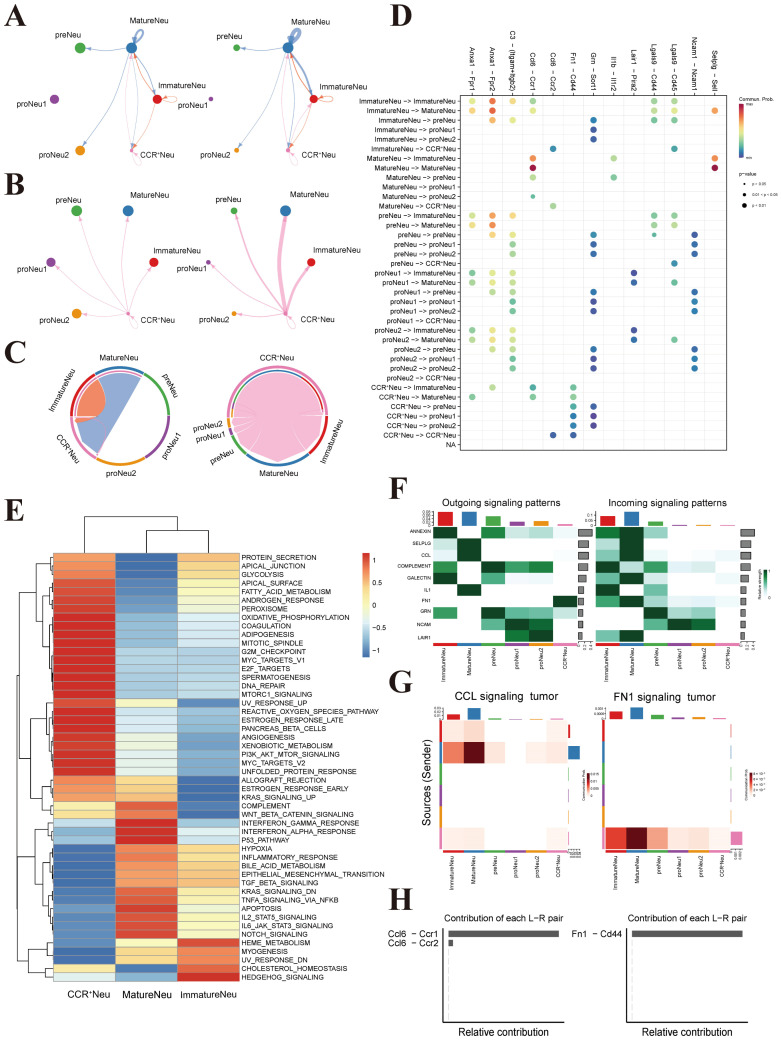
**CellChat- and GSEA-inferred intercellular communication among neutrophil states.** (**A**) Inferred CCL signaling networks for model and control groups; (**B**) inferred FN1 signaling networks for model and control groups; (**C**) inferred CCL and FN1 networks among neutrophil states in the model group; (**D**) significant inferred ligand–receptor pairs contributing to CCL and FN1 signaling; (**E**) GSEA HALLMARK analysis of CCR2^+^ neutrophil-like population, ImmatureNeu, and MatureNeu; (**F**) relative intensity of incoming and outgoing signaling across neutrophil states; (**G**) sender importance of each neutrophil state by network centrality in CCL and FN1 networks; and (**H**) relative contribution of each CCL and FN1 ligand–receptor pair to the overall signaling network. GSEA, Gene Set Enrichment Analysis; FDR, false-discovery rate.

CellChat inference of ligand–receptor communication (Figure 3D) linked the CCR2^+^ neutrophil-like population with mature neutrophils through two interactions. *Ccl6* highly expressed by ImmatureNeu and MatureNeu was inferred, based on the CellChatDB annotations, to signal to the CCR2^+^ neutrophil-like population via Ccr1 and Ccr2; given that CCR2 is canonically a CCL2 receptor for monocytes and macrophages, an inferred neutrophil response to CCL6 within the CD11b^+^Gr1^+^ compartment is noteworthy. Reciprocally, FN1 from the CCR2^+^ neutrophil-like population was inferred to engage CD44 on MatureNeu. Both pathways were more active in the tumor group (Figure 3A–C), with CCL signaling mediated by CCL6-CCR1 and CCL6-CCR2 and FN1 signaling mediated predominantly by FN1-CD44. Thus, inference positioned this population as signaling to MatureNeu chiefly via FN1-CD44 while receiving CCL6 signals in return (Figure 3F–H). HALLMARK comparison among the three states (Figure 3E) showed that this population upregulated G2M_CHECKPOINT, REACTIVE_OXYGEN_SPECIES_PATHWAY, ANGIOGENESIS, and PI3K_AKT_MTOR_SIGNALING, whereas MatureNeu upregulated inflammatory programs (INFLAMMATORY_RESPONSE and transforming growth factor-β, interleukin-, and interferon-related pathways) and tumorigenesis-related programs (EPITHELIAL_MESENCHYMAL_TRANSITION, P53_PATHWAY, and NOTCH_SIGNALING). As *Fn1* and *Cd44* are key EMT-associated genes, these patterns are consistent with the inference that the CCR2^+^ neutrophil-like population may be transcriptionally linked to MatureNeu.

### 3.5. The CCR2^+^ Neutrophil-like Population Is Not Recovered in Microbial Infection

To test whether a CCR2^+^ neutrophil-like population would also arise under cancer-unrelated inflammation, we analyzed GSE137539 (bone marrow Gr1^+^ cells from *E. coli*-infected mice), with GSM4081549/GSM4081550 serving as an infection group and GSM4081545/GSM4081546 serving as a control, and for visualization, we used the annotations of Xie et al. (Figure S1A,B). After isolating MP and neutrophils for Monocle analysis with the same markers (Figure S1C,D), we observed that none of the four groups showed a CCR2^+^ neutrophil branch or elevated *Ccr2*, and neutrophil proportions did not differ between groups (Figure S1E). The CCR2^+^ neutrophil state was therefore not recovered in this infection setting, suggesting its emergence is not a generic feature of inflammatory granulopoiesis.

### 3.6. Bulk Transcriptomics Links the Circulating Compartment to JAK-STAT and TGF-β

**Figure 4:**
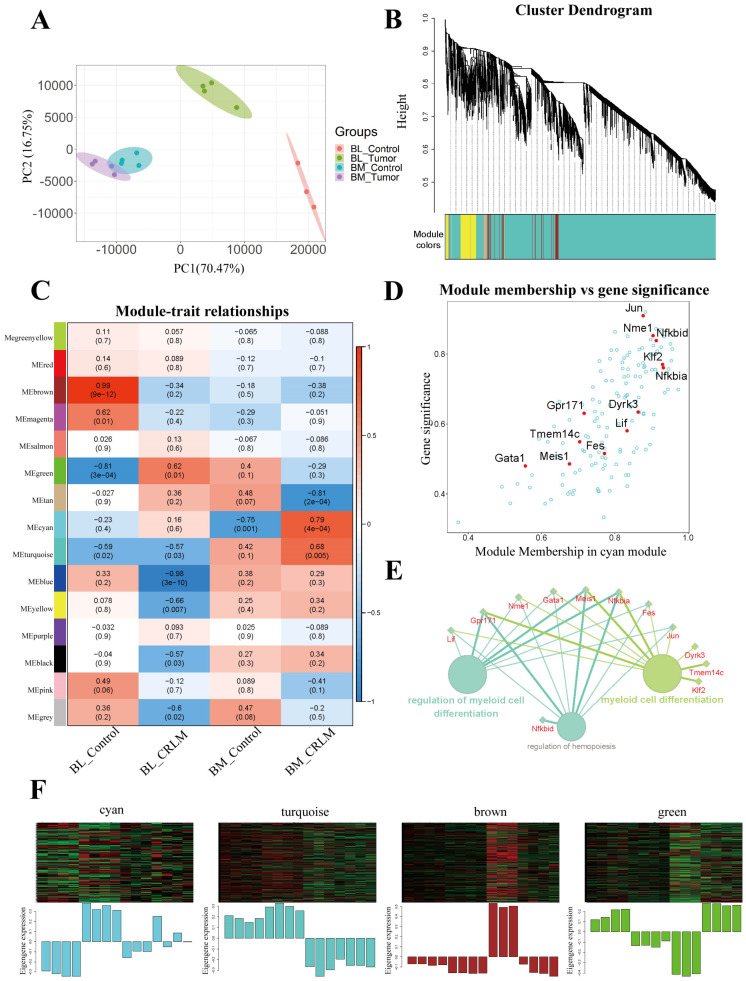
**PCA and WGCNA network construction for bulk RNA-seq of bone marrow and peripheral blood.** (**A**) PCA of the 15 bulk RNA-seq samples, showing separation among BM_CRLM, BL_CRLM, and BL_Control groups; (**B**) module dendrogram (1-TOM), with modules indicated by color; (**C**) module–trait associations between module eigengenes and sample groups; (**D**) gene significance versus module membership for BM_CRLM in the cyan module with hub genes; (**E**) GO enrichment of cyan-module hub genes for immune system processes (Fisher’s exact test, *p* < 0.05); and (**F**) eigengene heatmaps of the cyan, turquoise, brown, and green modules across all samples. BM_CRLM, bone marrow from CRLM mice; BL_CRLM, peripheral blood from CRLM mice; BL_Control, peripheral blood from control mice; MM, module membership; TOM, topological overlap matrix.

We performed bulk RNA sequencing and WGCNA on 15 samples (CRLM: 4 bone marrow, 4 blood; sham: 3 bone marrow, 4 blood; Figure 4A and Figure S3). A soft-threshold power of 8 achieved a scale-free topology (R^2^ = 0.85—Figure S2A,B), and dissTOM hierarchical clustering with dynamic tree cutting partitioned genes into 15 co-expression modules (Figure 4B, Figure S2C,D). Module–phenotype correlation (Figure 4C) identified MEcyan, MEturquoise, and MEgreen as significantly CRLM-associated. MEcyan genes were enriched in myeloid cell differentiation (GO immune-system process (Figure 4D,E)) and included neutrophil-differentiation transcription factors *Gata1*, *Jun*, and *Klf2*. Eigengene expression of the four most relevant modules (MEturquoise *p* < 0.1; MEcyan, MEbrown, MEgreen *p* < 0.01) is shown in Figure 4F, with module–sample relationships given in Figure S2E.

**Figure 5:**
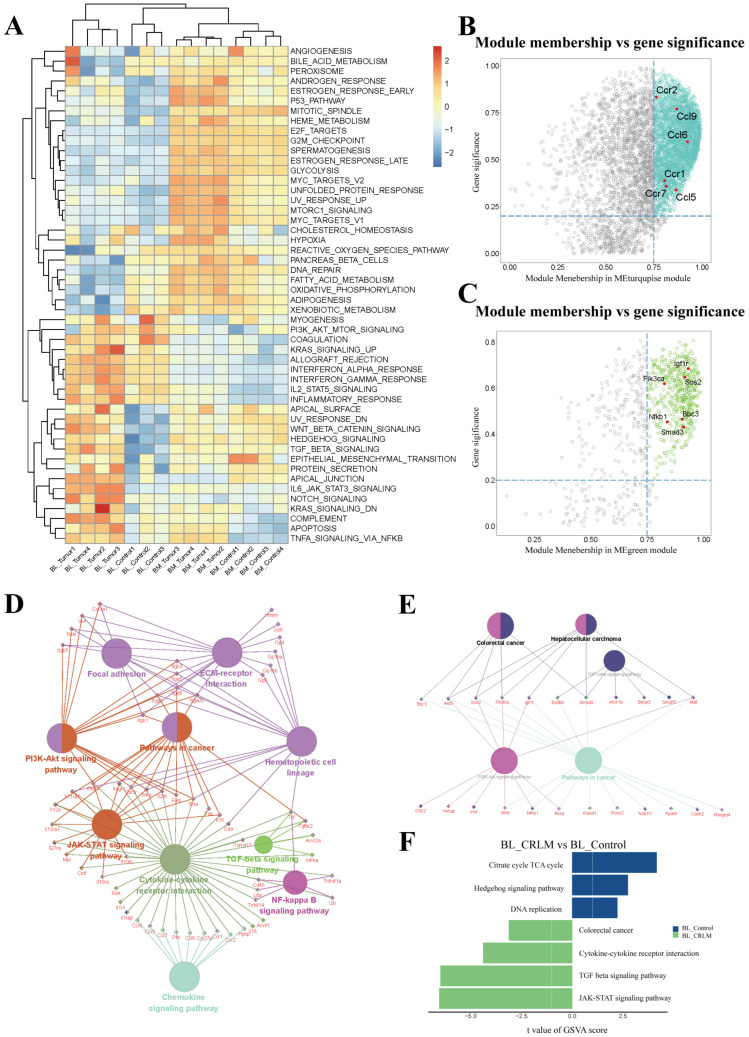
**WGCNA hub-gene and pathway analysis of bone marrow and peripheral blood.** (**A**) GSEA HALLMARK analysis of the 15 bulk RNA-seq samples comparing BM_CRLM with controls; (**B**) gene significance versus MM for BM_CRLM in the turquoise module, with hub genes (|MM| > 0.75, |GS| > 0.2) highlighted; (**C**) gene significance versus module membership for BL_CRLM in the green module, with hub genes (|MM| > 0.75, |GS| > 0.2) highlighted; (**D**) KEGG enrichment of turquoise-module hub genes (Fisher’s exact test, *p* < 0.05); (**E**) GO enrichment of green-module hub genes (Fisher’s exact test, *p* < 0.05); (**F**) and differential KEGG pathway activities stratified by GSVA between BL_Control and BL_CRLM (Benjamini–Hochberg adjusted *p* < 0.05).

Hub-gene analysis (|MM| > 0.75, |GS| > 0.2) showed that MEturquoise comprised chemokine members (*Ccr1*, *Ccr2*, *Ccl5*, *Ccl6*, *Ccl7*, and *Ccl9*) and integrins (*Itga2*, *Itga2b*, *Itga5*, and *Itga6*, and *Itgb1*, *Itgb3*, and *Itgb5*) (Figure 5B,D), enriched in cytokine–cytokine receptor interaction (mmu04060), JAK-STAT (mmu04630), ECM–receptor interaction (mmu04512), and TGF-β signaling (mmu04350) (Figure 5A,D). The MEgreen module of CRLM blood included *Igf1r*, *Pik3ca*, *Sos2*, *Bbc3*, *Nfkb1*, and *Smad3* (Figure 5C,E), enriched in cancer-associated pathways (Pathways in Colorectal Cancer mmu05210, Hepatocellular Carcinoma mmu05225). Consistent with the single-cell findings, CRLM blood hub genes converged on JAK-STAT (mmu04630) and TGF-β (mmu04350) signaling, both implicated in tumor metastasis (Figure 5F) [38,39].

### 3.7. Flow-Cytometric Validation of CCR2^+^ Neutrophil-like Population Enrichment in the Bone Marrow of CRLM Mice

**Figure 6:**
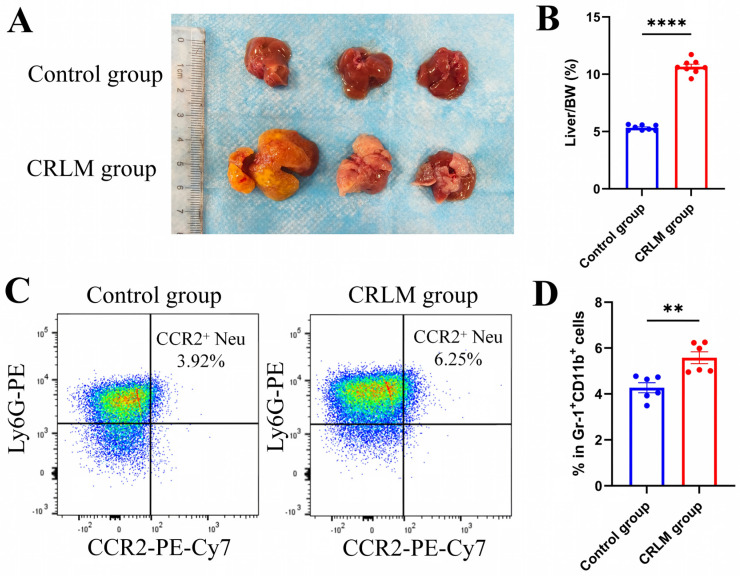
**Establishment of the MC38 CRLM model and flow-cytometric validation of CCR2^+^ neutrophils-like population.** (**A**) Representative liver images and (**B**) quantification of the liver/body weight ratio in control and CRLM mice (*n* = 8 mice/group). (**C**) Representative flow cytometry plots showing the gating of CCR2^+^ neutrophils within the CD45^+^CD11b^+^Gr1^+^ bone marrow myeloid compartment. (**D**) Quantification showed an increased frequency of CCR2^+^ neutrophils in CRLM mice (*n* = 6 mice/group). Data are shown as means ± SEMs. Statistical significance was determined by *t*-test. **, *p* < 0.01; ****, *p* < 0.0001.

To establish the MC38 colorectal liver metastasis (CRLM) model, mice underwent intrasplenic injection of MC38 cells followed by splenectomy, with control mice receiving PBS. On day 14 after injection, gross liver images showed visible tumor lesions in MC38-inoculated mice, whereas no tumor lesions were observed in the PBS-treated splenectomy controls (Figure 6A). Consistently, the liver/body weight ratio, used as an indicator of hepatic tumor burden, was significantly increased in the CRLM group compared with the control group (*p* < 0.0001), further confirming successful model establishment and tumor-associated liver enlargement (Figure 6B). To validate the scRNA-seq-identified CCR2^+^ neutrophil population, flow cytometry was performed using the CD45^+^CD11b^+^Gr1^+^ myeloid compartment as the parent gate, consistent with the sorting strategy used for scRNA-seq. The gating strategy can be found in Figure S4. Although CCR2^+^ neutrophil-like populations were detected in both the control and CRLM mice, the proportion was significantly higher in the CRLM mice than in the controls (*p* = 0.0031), supporting the enrichment of the CCR2-expressing neutrophil-like population during CRLM progression (Figure 6C,D).

## 4. Discussion

In this study, we used single-cell RNA sequencing to characterize the heterogeneity of bone marrow CD11b^+^Gr1^+^ myeloid cells in a murine model of colorectal liver metastasis. We identified a CCR2^+^ neutrophil-like subset enriched in CRLM-bearing mice, characterized by a CD11b^+^Gr1^+^Ly6G^hi^ phenotype and expression of *Ccr2*, *Mpo*, *Ctsg*, and *Fn1*. Trajectory and RNA velocity analyses placed this population along the neutrophil differentiation continuum and suggested that it represents an alternative terminal neutrophil state rather than a conventional monocytic population. Functionally, the CCR2^+^ neutrophil-like population displayed transcriptional programs related to cell-cycle activity, protein biosynthesis, reactive oxygen species, angiogenesis, and PI3K-AKT-mTOR signaling. Cell–cell communication analysis further predicted reciprocal crosstalk between the CCR2^+^ neutrophil-like population and mature neutrophils, comprising inward CCL6-CCR1/CCR2 signaling and outward FN1-CD44 signaling toward MatureNeu. In parallel, bulk transcriptomic analysis of the circulating compartment linked CRLM-associated gene modules to JAK-STAT, ECM–receptor interaction, and TGF-β signaling. Together, these findings nominate the CCR2^+^ neutrophil-like population as a previously underappreciated CRLM-associated myeloid population and provide a transcriptomic framework for future functional studies.

These observations extend the traditional monocyte-centered view of CCR2 biology in cancer. Once thought to be largely restricted to monocytes, CCR2 is increasingly recognized as functionally relevant to neutrophils as well [40]. It can mediate the mobilization of both monocytes and neutrophils from the bone marrow into peripheral blood [41,42], contributes to neutrophil accumulation in the joints of rheumatoid arthritis patients [25], and has been implicated in the recruitment of neutrophils to inflamed and metastatic tissues [43,44]. Here, we present a computational, single-cell-based report of a CCR2^+^ neutrophil-like subset in the bone marrow of CRLM mice associated with metastatic progression and liver colonization. Whereas prior work has established that CCR2^+^ monocytes are recruited to primary and metastatic sites, where they differentiate into immunosuppressive TAMs associated with poor prognosis in CRC [21,22,23], the potential role of the CCR2^+^ neutrophil-like population in CRC liver metastasis has remained largely unexplored. Our data suggest that, in CRLM, CCR2 expression is not confined to the monocytic compartment; it also marks a distinct neutrophil-like state potentially associated with metastatic niche remodeling and liver colonization. Notably, bulk RNA sequencing showed decreased *Ccr2* in bone marrow but increased *Ccr2* in peripheral blood from CRLM mice relative to controls. Because *Ccr2* is commonly interpreted as a monocyte-associated transcript in myeloid analyses, this divergence prompted us to examine whether a non-monocytic CCR2-expressing population could contribute to the circulating CCR2 signal in CRLM.

The identity of this population is supported by both marker expression and trajectory analyses. Although *Ccr2* expression in this subset was modest relative to monocytes, consistent with the low and inducible expression reported in non-tumor settings [26], the cluster was transcriptionally defined by neutrophil-associated genes including *Ly6g*, *S100a8/9*, and *Retnlg* rather than monocyte-lineage genes such as *Ly6c2* and *Csf1r*, a finding arguing against a simple monocyte-contamination artifact. Moreover, pseudotime and RNA velocity analyses positioned the CCR2^+^ neutrophil-like population within the neutrophil differentiation continuum, where it was inferred to emerge near terminal differentiation and represent an alternative terminal state in CRLM-bearing mice. These findings suggest that tumor-associated granulopoiesis may generate a CCR2-expressing neutrophil state distinct from conventional mature neutrophils.

Cell–cell communication inference further suggested that CCR2^+^ neutrophil-like populations may participate in bidirectional signaling with mature neutrophils. CCL6 from ImmatureNeu and MatureNeu was predicted to signal to the CCR2^+^ neutrophil-like population via CCR1 and CCR2, whereas FN1 from this population was inferred to engage CD44 on MatureNeu. Because this inference is based on ligand–receptor expression rather than functional perturbation, the FN1-CD44 pair should be interpreted as a predicted communication axis. Nevertheless, its association with EMT-associated transcriptional programs raises the possibility that crosstalk between neutrophil subsets may contribute to extracellular matrix remodeling in the metastatic niche. In peripheral blood bulk RNA sequencing, the disparity in *Il1b* and *Tgfb1* between blood and bone marrow became more pronounced, consistent with a circulating pro-inflammatory and pro-metastatic program. Across the 15 bulk samples, HALLMARK enrichment indicated that tumor-associated programs were more strongly enriched in peripheral blood than in bone marrow, including EMT and TGF-β signaling, a pattern corroborated in KEGG analysis. Relative to sham blood, CRLM blood showed upregulation of EMT-associated programs, encompassing the JAK-STAT (mmu04630) and TGF-β (mmu04350) signaling pathways, together with the colorectal cancer (mmu05210) and cytokine–cytokine receptor interaction (mmu04060) pathways. Together, the single-cell and bulk data showed that circulating inflammatory and matrix-remodeling programs are associated with CRLM progression.

Neutrophils are a central component of innate immunity, and disruption of homeostasis by tissue injury or tumor development triggers their mobilization from the bone marrow [27]. After a transient circulating phase, neutrophils are recruited to affected tissues in response to chemokines released by neoplastic cells, macrophages, and fibroblasts [45], and at metastatic sites, they display functional plasticity and adopt context-dependent phenotypes shaped by their polarization states and the TIME [46]. In this study, the CRLM-associated circulating program was enriched for genes linked to metastasis and progression, including *Smad3*, *Pik3ca*, and *Nfkb1*, and module hub genes converged at pathways related to metastasis, tumorigenesis, inflammation, and chemokine signaling. Notably, the CCR2^+^ neutrophil-like population was detected only in the MC38 CRLM model. To assess whether this subset was a generic feature of granulopoietic stress, we examined a publicly available dataset of bone marrow Gr1^+^ cells from *E. coli*-infected mice (GSE137539) [13], and an equivalent CCR2^+^ neutrophil population was not recovered, suggesting that this subset is not simply a product of microbial infection or general inflammation. Consistent with disease-context specificity, B16F10 melanoma liver metastasis does not recruit CD11b^+^Gr1^+^ subsets [14]. These findings support the possibility that a CCR2^+^ neutrophil-like population preferentially emerges in the CRLM context. More broadly, they are consistent with the notion that CD11b^+^Gr1^+^ myeloid responses vary substantially across tumor types, inflammatory contexts, and metastatic models. Together, these observations suggest that the emergence of the CCR2^+^ neutrophil-population is preferentially associated with the CRLM context, and they add to the evidence that CC-chemokine receptors can be expressed by neutrophils rather than exclusively by monocytes and macrophages, adding a layer of complexity to chemokine-mediated regulation of metastasis.

We interpret the CCR2^+^ neutrophil-like population as a transitional or alternative terminal neutrophil state, distinct from both conventional mature neutrophils and classical granulocytic progenitors, with transcriptional features overlapping with those of PMN-MDSCs. We also observed elevated *Tgfb1* transcription in the CRLM group. PMN-MDSCs recruited to primary melanoma sites have been reported to promote EMT through HGF and TGF-β [47,48], and our inferred FN1-CD44-associated EMT program, accompanied by *Tgfb1* upregulation, is consistent with this paradigm, suggesting that the CCR2^+^ neutrophil-like population identified here may represent a granulocytic state related to PMN-MDSCs. TGF-β both polarizes tumor-associated neutrophils toward the pro-tumor N2 phenotype [46,49] and mediates EMT with fibronectin deposition, whereas TGF-β blockade abrogates N2 polarization yet can enhance CD11b^+^Gr1^+^ recruitment in breast cancer [15], underscoring that the TGF-β-CD11b^+^Gr1^+^ relationship is reciprocal and context-dependent. Whether circulating tumor-associated neutrophils are genuinely derived from PMN-MDSCs remains unresolved [50]. Overall, our transcriptome-based findings are consistent with a model in which neutrophils may adopt an immunosuppressive and pro-tumor phenotype associated with TGF-β signaling and are transcriptionally linked to Tgfb1- and Fn1-related inflammatory and matrix-remodeling programs, potentially involving Cd44-expressing mature neutrophils in EMT-related processes. However, this model requires direct functional testing.

These findings carry potential clinical and research implications. The identification of a CRLM-associated CCR2^+^ neutrophil-like population suggests that CCR2-targeted strategies currently under clinical evaluation for monocyte-directed immunotherapy may warrant re-examination in the context of neutrophil biology. From a research perspective, the transcriptional programs identified here, particularly the FN1-CD44 axis and TGF-β-associated signatures, provide candidate targets and mechanistic hypotheses for future functional studies, including lineage-tracing, adoptive transfer, and in vivo perturbation experiments in CRLM models.

This study has several limitations. Our findings are primarily derived from single-cell and bulk transcriptomic inference, and therefore the proposed identity of the CCR2^+^ neutrophil-like population, the FN1-CD44/EMT interaction, and the JAK-STAT/TGF-β-associated programs require validation at the protein, functional, and in vivo levels. The identity of this population is provisionally interpreted as a transitional neutrophil state with PMN-MDSC-like transcriptional features pending functional and protein-level validation. In addition, the modest *Ccr2* expression levels and the inherent dropout of scRNA-seq further warrant confirmation of surface CCR2 expression on Ly6G^+^ cells. Furthermore, the scRNA-seq analysis was based on a single animal per group, limiting inter-individual variability assessment; the WGCNA network was constructed from 15 samples at the minimum recommended threshold; and no external CRLM bone marrow single-cell datasets were available for independent validation. Despite these limitations, our study provides transcriptomic evidence of a previously underappreciated CRLM-associated CCR2^+^ neutrophil-like population and links this subset to neutrophil differentiation, intercellular communication, and circulating pro-metastatic signaling programs. These findings broaden the current monocyte-centered view of CCR2 biology and provide a foundation for future mechanistic studies targeting neutrophil-mediated metastatic progression.

## 5. Conclusions

In summary, integrated single-cell and bulk transcriptomic analysis identified a previously underappreciated CCR2^+^ neutrophil-like subset enriched in the bone marrow of MC38 intrasplenic-injection CRLM mice. This subset was positioned along the neutrophil differentiation trajectory and was associated with inferred FN1-CD44 signaling with mature neutrophils, together with upregulation of EMT-related genes such as *Il1b*, *Tgfb1*, and *Cd44* and with JAK-STAT and TGF-β programs. Given the established role of PMN-MDSCs in inducing EMT and metastasis, this CCR2^+^ neutrophil-like population may represent a CRLM-associated granulocytic state with potential pro-metastatic relevance. Our findings suggest that the CCR2-expressing cells associated with CRLM may include neutrophils rather than monocytes alone and that this subset appears preferentially associated with the CRLM context. Although these transcriptome-derived associations remain hypothesis-generating and warrant future functional and in vivo validation, our study provides a framework for future investigation of neutrophil-mediated metastatic niche formation in CRLM.

## Data Availability

The raw data generated in this study have been deposited in the Genome Sequence Archive of the National Genomics Data Center (NGDC) under accession number CRA020639 (https://ngdc.cncb.ac.cn/gsa). Access to the data is subject to approval by the data access committee on reasonable request. The extra validation datasets of mice without CCR2^+^ neutrophils were downloaded from the Gene Expression Omnibus (GEO) database (GSE137539). No custom code or software was generated in this work.

## Supplementary Materials

The following supporting information can be downloaded at https://www.mdpi.com/article/10.3390/genes17070831/s1. Figure S1: Comparison of bone marrow Gr1^+^ lineages in an external microbial-infection dataset (GSE137539). Figure S2: Soft-threshold selection and module characterization in the WGCNA network. Figure S3: Differential gene expression in bone marrow and peripheral blood determined by bulk RNA sequencing. Figure S4. Gating strategy for identification of CCR2^+^ neutrophils in bone marrow by flow cytometry.

## Abbreviations

The following abbreviations are used in this manuscript:

CRLM	Colorectal liver metastasis
scRNA-seq	Single-cell RNA sequencing
UMAP	Uniform manifold approximation and projection
DEGs	Differentially expressed genes
GO	Gene ontology
GSEA	Gene set enrichment analysis
PCA	Principal component analysis
PCs	Principal components
WGCNA	Weighted correlation network analysis
TOM	Topological overlap matrix
MM	Module membership
GS	Gene significance
KEGG	Kyoto Encyclopedia of Genes and Genomes
GSVA	Gene set variation analysis
