# Supplementary material for: Single-Cell Profiling Identifies a CCR2+ Neutrophil-like Population Associated with Colorectal Cancer Liver Metastasis in a Murine Model
Source: Genes (Basel). 2026 Jul 21;17(7):831. doi: 10.3390/genes17070831 (PMC13409406; doi:10.3390/genes17070831)
Supplement: Supplementary file 1 [file genes-17-00831-s001.zip › genes-4407219-supplementary.pdf]

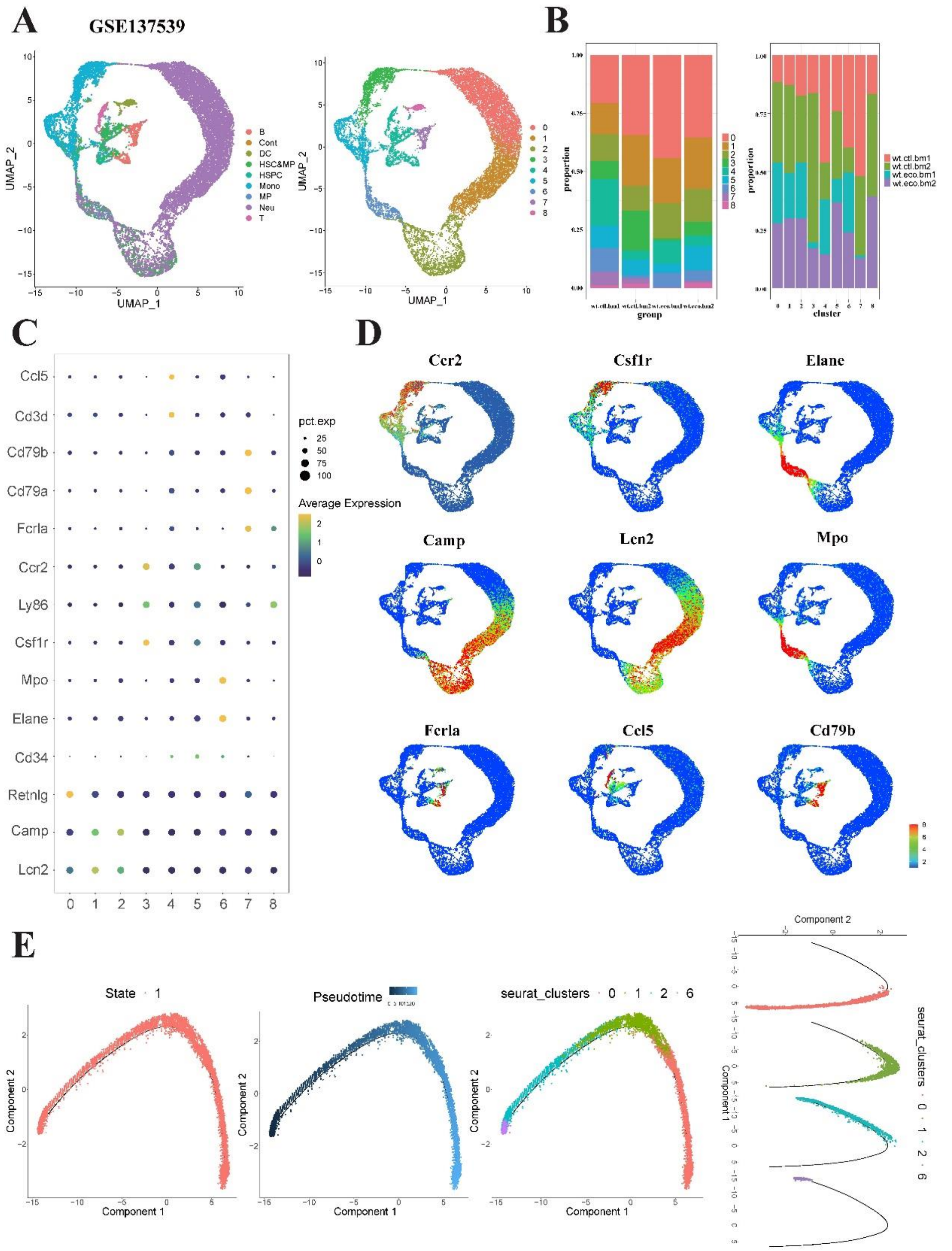

Figure S1. Comparison of bone marrow Gr1⁺ lineages in an external microbial-infection dataset (GSE137539). (A) UMAP of bone marrow cells from *E. coli*-infected and control mice colored by annotated cell type (left) and by cluster (right); (B) Proportion of clusters per sample (left) and proportion of samples per cluster (right); (C) Dot plot of canonical marker gene expression across clusters; (D) UMAP feature plots of *Ccr2*, *Csf1r*, *Elane*, *Camp*, *Lcn2*, *Mpo*, *Fcrla*, *Ccl5*, and *Cd79b*; (E) Monocle pseudotime trajectory of myeloid progenitors and

neutrophils, colored by state (left), pseudotime (middle), and Seurat cluster (right), with branch structure shown separately. wt.ctl, control mice; wt.eco, *E. coli*-infected mice; bm, bone marrow.

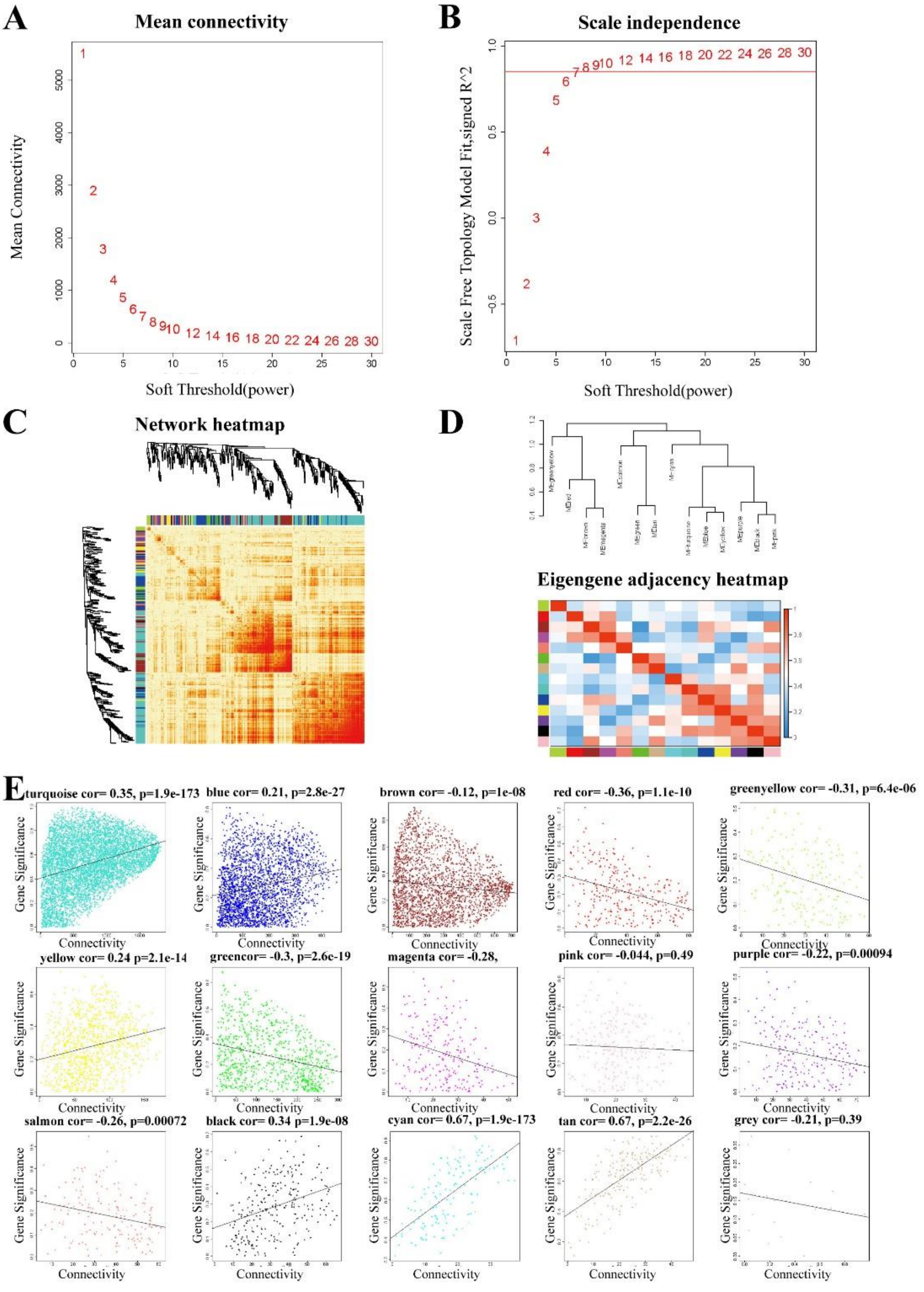

Figure S2. Soft-threshold selection and module characterization in the WGCNA network. (A) Mean connectivity across candidate soft-threshold powers; (B) Scale-free topology model fit (signed R^2) across candidate soft-threshold powers, with a power of 8 selected at the 0.85 threshold; (C) Network heatmap of topological overlap among genes, with the gene dendrogram and module color assignments; (D) Hierarchical clustering of module eigengenes (top) and eigengene adjacency heatmap (bottom); (E) Scatter plots of gene significance versus

intramodular connectivity for all modules, with Pearson correlation coefficient and p-value indicated for each module.

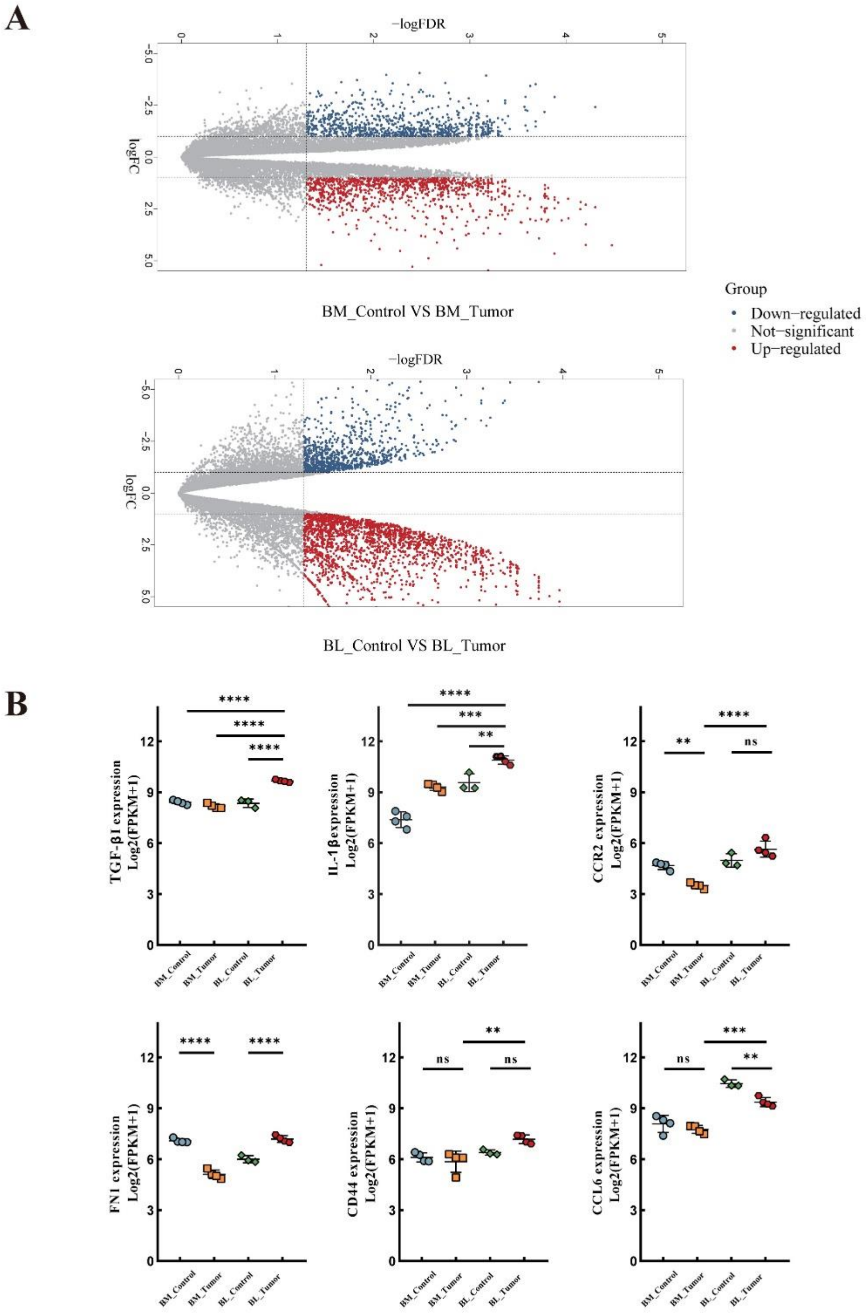

Figure S3. Differential gene expression in bone marrow and peripheral blood by bulk RNA sequencing. (A) Volcano plots of DEGs between control and CRLM groups in bone marrow (BM_Control vs BM_CRLM, top) and peripheral blood (BL_Control vs BL_CRLM, bottom), with upregulated genes in red, downregulated genes in blue, and non-significant genes in grey (adjusted $p < 0.05$, $|\log_2 FC| > 1.25$); (B) Expression of *Tgfb1*, *Il1b*, *Ccr2*, *Fn1*, *Cd44*, and *Ccl6* ($\log_2$(FPKM + 1)) across the BM_Control, BM_CRLM, BL_Control, and BL_CRLM groups (two-tailed unpaired Student's t-test; ns, not significant; $^{**}p < 0.01$; $^{***}p < 0.001$; $^{****}p < 0.0001$).

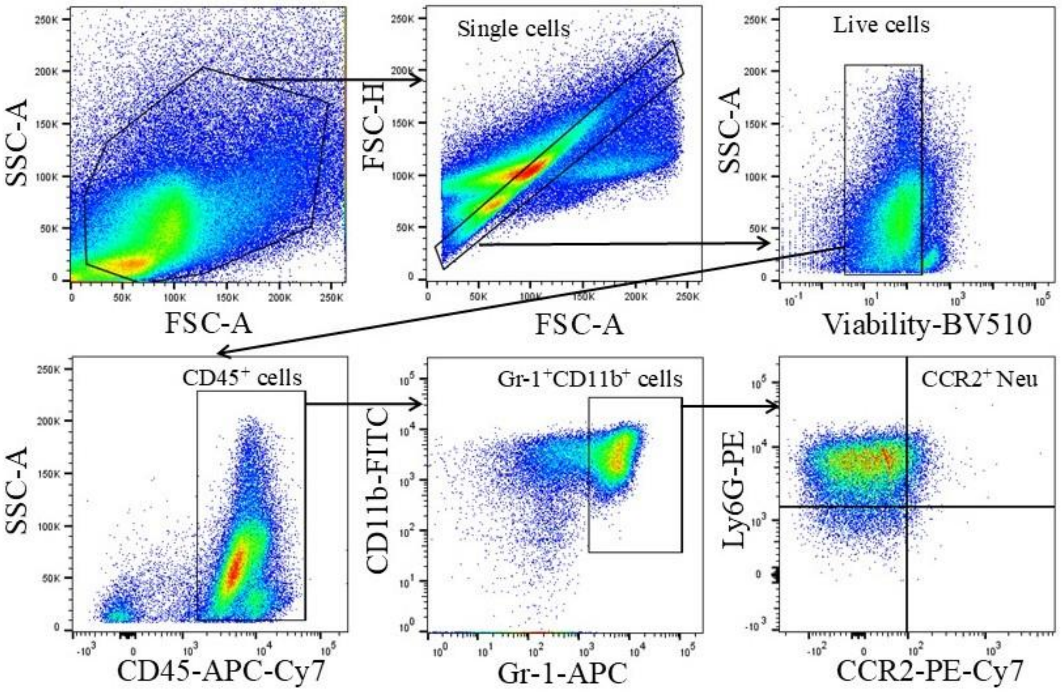

Figure S4. Gating strategy for identification of CCR2⁺ neutrophils in bone marrow by flow cytometry. Representative flow cytometry plots showing the sequential gating strategy applied to bone marrow cells from CRLM and control mice.
